# The Exploration of Microbial Natural Products and Metabolic Interaction Guided by Mass Spectrometry Imaging

**DOI:** 10.3390/bioengineering9110707

**Published:** 2022-11-18

**Authors:** Hao Li, Zhiyong Li

**Affiliations:** State Key Laboratory of Microbial Metabolism, School of Life Sciences and Biotechnology, Shanghai Jiao Tong University, Shanghai 200240, China

**Keywords:** mass spectrometry imaging, microorganism, natural products, metabolic interaction

## Abstract

As an impressive mass spectrometry technology, mass spectrometric imaging (MSI) can provide mass spectra data and spatial distribution of analytes simultaneously. MSI has been widely used in diverse fields such as clinical diagnosis, the pharmaceutical industry and environmental study due to its accuracy, high resolution and developing reproducibility. Natural products (NPs) have been a critical source of leading drugs; almost half of marketed drugs are derived from NPs or their derivatives. The continuous search for bioactive NPs from microorganisms or microbiomes has always been attractive. MSI allows us to analyze and characterize NPs directly in monocultured microorganisms or a microbial community. In this review, we briefly introduce current mainstream ionization technologies for microbial samples and the key issue of sample preparation, and then summarize some applications of MSI in the exploration of microbial NPs and metabolic interaction, especially NPs from marine microbes. Additionally, remaining challenges and future prospects are discussed.

## 1. Introduction

Bioactive microbial natural products (NPs) are important sources of leading drugs [1]. Since the discovery of penicillin [2], human exploration in the field of microbial NPs has continued for nearly 100 years. To date, microbial natural products and their derivatives have made a great contribution to human health, especially for antitumor and antimicrobial drug development [3]. Generally, in a routine microbial NPs’ research workflow, after the isolation, NPs need to be characterized by several methods for their chemical properties. As a regular analytic and characteristic technology, mass spectrometry is an indispensable tool in this field, which offers qualitative (mass-to-charge ratio) and quantitative (intensity) information for specific molecules [4,5]. However, traditional mass spectrometry analysis for microbial NPs requires laborious processes including fermentation, extraction, concentration and dissolution, which often cost days to weeks. More importantly, the compounds are removed from their original biological environment, which prevents the understanding of NPs’ real biofunctions or biosynthetic origins in site [6].

At the forefront of microbial NPs’ discovery, different kinds of advanced mass spectrometry imaging (MSI) technologies enable direct characterization and analysis of microbial NPs or microbial interaction, which allows researchers to connect the microbial phenotypic phenomena to chemical information [7]. As MSI can capture the spatial distribution of the chemical information of analytes, it combines the mass spectrum data and molecules’ spatial distribution into one experiment, which cannot be achieved by traditional mass spectrometry.

**Figure 1:**
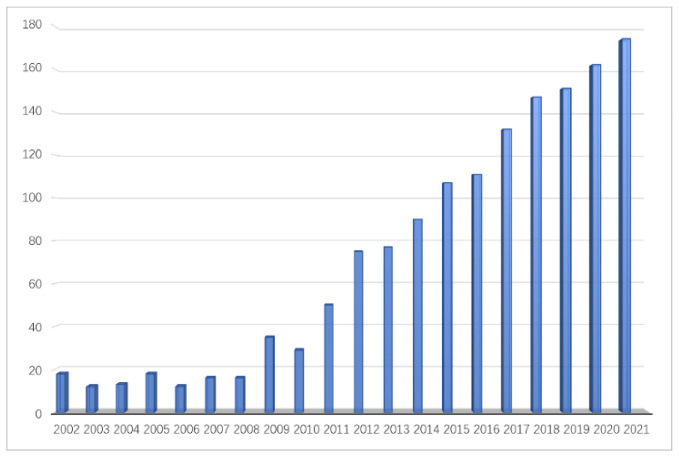
Microbial mass spectrometry imaging-related papers on Web of Science™ from 2002–2021.

MSI initially began with solid surface analysis with secondary ion mass spectrometry (SIMS) imaging in the early 1960s, and progressed with commercial laser microprobe mass spectrometry (LMMS) [8]. In recent years, largely benefitting from the advances in the soft ionization technique, MSI has been applied rapidly in the biology field [7], since the investigation of peptides and proteins in biological samples based on matrix-assisted laser desorption/ionization (MALDI-TOF-MS) [9]. The research of microbial MSI has continued to grow in the past two decades (Figure 1). With the ability of direct untargeted investigation of diverse molecules such as glycans, lipids, peptides and macromolecules, with a label-free method on a complex biological sample surface, MSI offers a powerful tool to complement other biological imaging and omics study [10].

**Figure 2:**
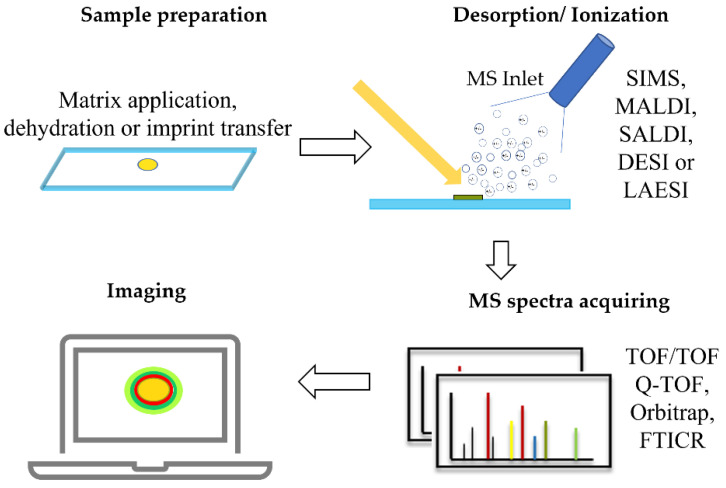
A typical workflow of microbial MSI. SIMS: secondary ion mass spectrometry; MALDI: matrix-assisted laser desorption/ionization; SALDI: surface-assisted laser desorption/ionization; DESI: desorption electrospray ionization; LAESI: laser ablation electrospray ionization; TOF: time-of-flight, Q-TOF: quadrupole time-of-flight, FTICR: Fourier transform ion cyclotron resonance.

As shown in Figure 2, a standard MSI workflow generally contains four steps: (1) the sample is induced to desorption under the laser or ion beam gun; (2) the gas phase is ionized for further detection in the mass analyzer (please find the detailed ionization strategy in Table 1); (3) the detector records the values of the mass-to-charge ratio (m/z) and generates a mass spectrum graph, with m/z values on the *x* axis and ion intensity on the *y* axis; and (4) the collected mass spectra of every pixel on the surface of the analytes are performed as a heat map to visualize the spatial chemical information using professional data processing software [11].

It is worth noting that the application of MSI in microbial NPs’ research has led to great achievements [7,11,12,13,14,15,16,17,18,19,20,21,22]. With the aim to attract more attention from microbial NPs researchers to this impressive technology and inspire more applications in this field, this review mainly focuses on the impressive applications of MSI in microbial NPs in recent years, including microbial monoculture, bipartite interaction and complex microbiome.

## 2. Different Ionization Used in Microbial MSI

For any mass spectrometry analysis, the ionization level and efficiency of the analytes should be considered as one of the most important factors [7,27]. To date, diverse commercial ion sources have been used for microbial MSI (Table 1). Generally, we divide them into two sections—“vacuum ionization” and “ambient ionization”—according to the ion source working condition [13]. Secondary ion mass spectrometry (SIMS), matrix-assisted laser desorption/ionization (MALDI), surface-assisted laser desorption/ionization (SALDI), desorption electrospray ionization (DESI) and laser ablation electrospray ionization (LAESI) are the five major MSI methods in the field of microbial NPs. Spraker et al. recently reviewed the ionizations in MSI for NPs’ research that can be used as a guide for those seeking further background reading [7].

### 2.1. Secondary Ion Mass Spectrometry (SIMS)

As the earliest MSI technology used for microbial research [28], SIMS depends on the charge transfer from the high-energy primary ion beam to the sample surface for generating the analyte secondary ions. SIMS can provide superior spatial resolution (<100 nm), far beyond other MSI technologies [29]. However, the harsh requirements for the surface of samples limit the application of SIMS in the exploration of microbial NPs. Cultivating microbial samples on conductive substrate surfaces such as silicon wafer and indium tin oxide-coated (ITO) glass slides can partly solve this problem [30,31]. On the other hand, due to the nature of the hard ionization technique, SIMS produces some poorly interpretable secondary fragmentation ions, which limits the application in the identification of unknown natural products [11]. As a means of optimization, matrix-enhanced SIMS (ME-SIMS) can partly help to reduce fragmentation, enhance ionization and extend the mass range of SIMS experiments [32].

### 2.2. Matrix-Assisted Laser Desorption/Ionization (MALDI)

In 1985, MALDI was firstly discovered by Franz Hillencamp and Michael Karas [33], and then Koichi Tanaka developed this technology into protein analysis and won the Nobel Prize in 2002 [34]. Due to the broad coverage of molecule species (small molecules, lipopeptides, peptides and proteins), MALDI has become the first choice of microbial MSI [35]. In MALDI-MSI, biomolecules on the sample surface are desorbed by the assistance of laser light and the matrix (small organic acids or bases that cocrystallize with the sample) for proper ionization [36]. Hence, the selection of a suitable matrix is considered as one of the critical factors, especially for NP research [37]. Until now, 2,5-dihydroxybenzoic acid (DHB), α-cyano-4-hydroxycinnamic acid (CHCA) and their mixture have been the most common choices for various small molecule imaging in positive ion mode [38], while in negative ion mode, 9-aminoacridine (9-AA) has shown the advantage of low background with MALDI-MSI [39]. In addition, 3,5-dimethoxy-4-hydroxycinnamic acid (SA, sinapinic acid) is used for protein analysis, and 4,6-Trihydroxyacetophenone (THAP) [40] and 3-Hydroxypicolinic acid (3-HPA) [41] are justified to be suitable for oligonucleotide analysis. In addition, many other matrices substrates such as Dithranol [42], Curcumin [43], 4-Phenly-α-cyanocinnamic acid amide [44], 1,6-diphenyl-1,3,5-hexatriene (DPH) [45], 3-Aminophthalhydrazide (Luminol) [38], N-phenyl-2-naphthylamine (PNA) [46], 1,5-diaminonapthalene (1,5-DAN) [47], 1-naphthylhydrazine hydrochloride (NHHC) [48] have been used. Meanwhile scientists are still searching for new matrices with better performance.

### 2.3. Surface-Assisted Laser Desorption/Ionization (SALDI)

SALDI-MS was originally proposed by Sunner and Chen in 1995 [49]. As a kind of matrix-free laser desorption/ionization (LDI) technology, SALDI substitutes the organic matrix of MALDI with other substrate surfaces such as graphite or nano silicon [7]. Due to the avoidance of organic matrices, SALDI provides the ideal prospect for application in small molecules (<500 Da) [50,51]. In the past decade, there were several applications of biological and microbial SALDI imaging based on different kinds of silicon substrates [52,53,54]. For instance, Wang et al. prepared gold nanoparticles/thiol-β-cyclodextrin-functionalized TiO_2_ nanowires as the assistant surface for NP SALDI-MSI in 2022 [50]. Meanwhile, several studies have indicated that SALDI could be a promising platform for microbial imaging [21,55].

### 2.4. Desorption Electrospray Ionization (DESI)

In 2004, as a novel ambient ionization technology, DESI was firstly innovated by Cooks’ group [56]. In a standard DESI process, the high-velocity electrosolvent is directly sprayed on the surface of the sample to form sample-bearing droplets, and then the ions generated by desolvation of these droplets are introduced to the mass spectrometer inlet [14]. Routinely, in order to obtain ideal imaging results, the optimization for instrument parameters is inevitable, such as solvent selection, solvent flow rate, spray voltage, gas flow rate, and the distances and angle from the sample surface to the sprayer [11]. However, DESI-MSI requires a hard, flat, uniform and nonconductive sample surface for the consistent and stable signal, which partly limits the application of DESI-MSI to microbial NP analysis [57].

### 2.5. Laser Ablation Electrospray Ionization (LAESI)

LAESI was invented in 2007 by Nemes and Vertes [58] and provided a new choice for ambient ionization mass spectrometry technology via the combination of electrospray ionization and laser ablation. Gas particles on the analyte surface, formed by mid-infrared laser ablation, are introduced to the MS inlet for analysis after electrospray ionization. LAESI requires little sample preparation but samples should be water-rich, which allows direct analysis of some limited sample surfaces such as plant tissues, clinical samples and microorganisms [59]. In 2016, Li et al. first reported the application of LAESI-MSI in a bacterial colony on agar to show the bacterial lipids’ distribution, which presented its ability in the identification and imagination of microbial metabolites [60].

## 3. Sample Preparation in Microbial MSI

Unlike usual vegetal or mammalian tissue section imaging, sample preparation for microbial samples in MSI is a tricky problem [21]. Flat frozen sections of organs or tissues can be easily obtained from cryogenic microtomes, but, unfortunately, microbial agar cultures are often too thin for cryotome sectioning. In addition, most microbial samples contain complex topography due to the presence of spores and aerial hyphae on the hydrated agar media, which makes it difficult to meet the requirements of the ion source for the analyte surface. For those vacuum ion sources such as MALDI, it demands a dry sample and homogeneous deposition of the specific matrix. Though it is a tough challenge, much useful and impressive progress has been made this decade, since the first protocol for agar-based microbial MALDI-MSI was introduced [61]. A typical microbial sample preparation contains three steps: (1) culturing the microbe on thin agar; (2) dehydrating the sample; and (3) depositing the matrix on the sample surface. Actually, the factors to be considered in each specific experimental process are far more complicated than the three steps above. There are many details to be optimized such as the agar concentration of the culture media, the choice of matrix and the matrix deposition methods (including sieving, sublimation, using an airbrush and a robotic sprayer) [21]. Some advances in the sample preparation of microbial MALDI-MSI are summarized in Table 2. It should be noticed that, due to the construction of the instrument and unstable sample adherence, the agar sample placed on the MALDI target plate may flake or even fall from the plate under a high-vacuum condition, which brings disadvantages to the experiment and even causes irreversible damage to the instrument. Cultivating microbes on a glass slide coated with indium tin oxide (ITO) or some optimization of the sample preparation can partly overcome this problem [35,61]. Meanwhile, AP-MALDI was subsequently developed and has been performed to monitor the spatial metabolome of the host–microbe associations of deep-sea mussel *Bathymodiolus puteoserpentis* at atmospheric pressure, which will alleviate this vacuum restriction problem [10,62].

DESI-MSI, as the other mainstream option used for NP or microbial metabolite imaging, requires relatively simple sample preparation compared to MALDI-MSI. Some technical advances in the sample preparation of microbial DESI-MSI are summarized in Table 3. Routine DESI-MSI analysis demands a hard, flat and uniform surface. Several viable alternative solutions have been developed, for example, imprint transfer technology, the involved metabolites and its distribution on the surface of the microbial culture medium is transferred to mixed cellulose ester filter membranes, and thus DESI analysis can be performed on this flat membrane [57]. However, this method might lose some molecular information and is not ideal for those microbial samples with complex surface conditions such as fungi [69]. Another simple but exciting advance in microbial sample preparation for DESI-MSI is dehydration. For microbes grown on a thin-layered agar plate, the extra dehydration can form a flat, hard and nonconductive surface, which is suitable for direct DESI-MSI analysis [69]. Nano-DESI-MSI technology has the ability of minimally invasive and direct analysis of a living microbial community, without any sample preparation [70].

## 4. Applications of MSI in Microbial NP Research

### 4.1. The Identification and Discovery of Microbial NPs

It is generally believed that in order to adapt to a complex stressful environment, microorganisms tend to produce some NPs with antipredation, antibacterial and antifungal activities. Now, visualized MSI can provide convincing evidence in the verification of this hypothesis. One of impressive cases is the discovery of arylomycin, a class of broad-spectrum antibiotics that target type I signal peptidases from *Streptomyces roseosporus,* which demonstrated that MSI can be a “bridge” to connect phenotypes with chemotypes [74]. In 2013, Moree et al. cocultured *Bacillus amyloliquefaciens* GA40, isolated from an octocoral in Panama, with two fungal strains, *Aspergillus fumigatus* and *Aspergillus niger*, and then they revealed the antifungal compound, iturin lipopeptide, using combined MALDI-MSI and MS/MS networking [75]. In 2015, using the integrated approach of MSI and molecular networking, a series of amino-polyketide derivatives and vitroprocines A-J were discovered from the marine bacterium *Vibrio* sp. QWI-06, with activity against *Acinetobacter baumannii* [76]. The latest exciting example is the biosynthetic potential of the *Arabidopsis* leaf microbiome. Helfrich et al. monitored over 50,000 binary interactions of 224 strains isolated from the *Arabidopsis* leaf and discovered three distinct natural product scaffolds with the combination of MADLI-TOF-MSI [77]. Some other attempts and contributions in this field of monocultured microbial NPs or metabolites are summarized in Table 4. Through an imaging study of 40 microbes, it is fully demonstrated that MALDI-MSI can be used as a general tool to study the NPs from diverse microbes [78].

### 4.2. The Localization and Searching for the Real Producer of Microbial NPs

**Figure 3:**
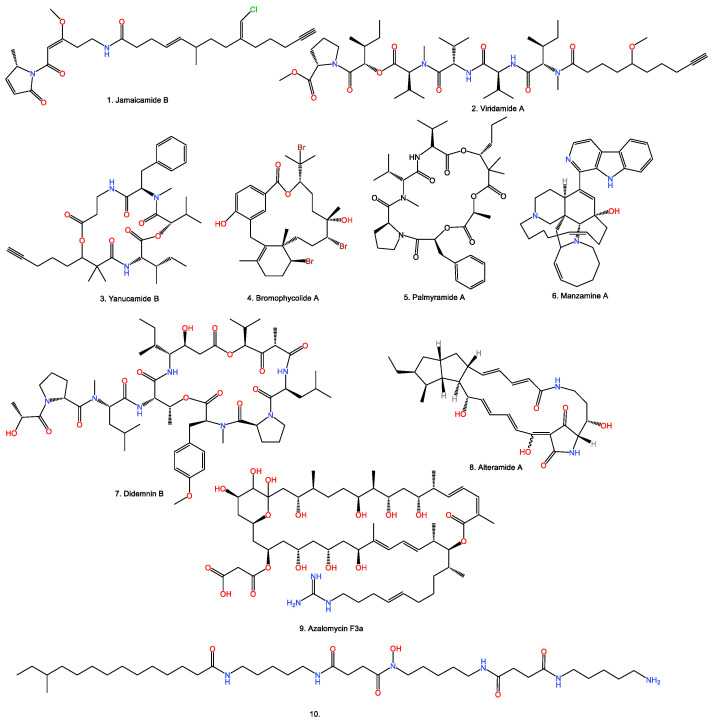
Some observed microbial NPs using MSI.

In the early stage of MSI applications in microbial NPs, researchers focused mainly on the localization and spatial distribution of specific NPs in mixed samples [79]. In 2008, Esquenazi et al. observed the location of marine NPs Jamaicamide B and Yanucamide B in single cyanobacterial filaments, while observing curazole, curacin, viridamides A and viridamides B in intact cyanobacteria (Figure 3) [79]. In 2009, Lane et al. measured the quantities and the location of antifungal natural products bromophycolids (Figure 3) on the surface of marine red alga *Callophycus serratus* using DESI-MSI, and demonstrated the antifungal chemical defense mechanism of *C. serratus* [80,81], which is hard to achieve by other techniques.

**Table 4:** MSI application in the research of monocultured microbial NPs or metabolites.

Species	Ionization Method	Compound	Research Purpose	Reference
*Actinomyces* sp. CNS 575	MALDI	Etamycin	Identification	[78]
*Beauveria bassiana* ATCC 7159	MALDI	Beauvericin, bassianalide	Identification	[78]
*Nostoc* sp.	MALDI	Pheophytin A	Identification	[78]
*Streptomyces coelicolor* A3	MALDI	SapB, CDA	Identification	[78]
*Fusarium* sp. CNL-292	MALDI	Sansalvamide	Identification	[78]
*Streptoverticillium griseoverticillatum* ATCC 31499	MALDI	Cinnamycin	Identification	[78]
*Bacillus subtilis* 3610	MALDI	Surfactin, plipastatin	Identification	[78]
*Lysobacter enzymogenes* C3	MALDI	Maltophilin, dihydromaltophilin	Identification	[78]
*Staphylococcus aureus*	MALDI	δ-toxin	Identification	[78]
*Candida albicans*	SIMS	Chlorhexidine digluconate (CHG)	Distribution of the CHG within the biofilms	[82]
*Streptomyces coelicolor*	SIMS	Undecylprodigiosin, butylcyclohexylprodiginin, actinorhodins	Antibiotic distribution on the cell surface	[83]
*Bacillus subtilis*	SIMS	Surfactins	Localization and quantification	[84]
*Streptomyces griseus* IFO 13350	MALDI	Lantipeptide AmfS	Discovery of new natural products	[85]
*Streptomyces hygroscopicus* ATCC 53653	MALDI	StendomycinⅠ-Ⅵ	Discovery of new natural products	[85]
*Streptomyces sviceus* ATCC 20983	MALDI	Lasso peptide SSV-2083	Discovery of new natural products	[85]
*Bacillus amyloliquefaciens* S499	SIMS	Surfactins, iturins and fengycins	Characterization of the interaction between cyclic lipopeptides with the plant	[86]
*Staphylococcus aureus* USA300 TCH1516	MALDI	Phenol-soluble modulins	Study the metabolism of community-associated methicillin-resistant *Staphylococcus aureus*	[87]
*Mycena metata*	MALDI	6-Hydroxymetatacarboline D	Discovery of new natural products	[88]
*Pseudomonas aeruginosa*	MALDI	*P. aeruginosa* derived metabolites	Microbial metabolite manner under the treatment of azithromycin	[89]
*Herpetosiphon* sp. B060	MALDI	Siphonazole	Biosynthetic pathway	[90]
*Microcystis aeruginosa* PCC 7820	MALDI	Aeruginosin 602, microcystin-LR	Distribution	[91]
*Nodularia. harveyana* PCC 7804	MALDI	Anachelin	Distribution	[91]
*Anabaena Cylindrica* PCC 7122	MALDI	Nodularin-R, nodularin-[Har]	Distribution	[91]
*Streptomyces* sp. Mg1	MALDI	Linearmycins	Localization	[92]
*Pseudomonas aeruginosa*	SIMS	Alkyl-quinolone	Quantitation	[93]
*Penicillium digitatum*	DESI	Indole alkaloids	Characterization and identification	[94]
*Streptomyces* sp. Caat 1-54	DESI	Lienomycin, lysolipin I	Characterization and identification	[95]

Note: DESI: desorption electrospray ionization; MALDI: matrix-assisted laser desorption/ionization; SIMS: secondary ion mass spectrometry.

Recently, increasing studies have reported that plant endophytic microorganisms can produce some NPs, originally known from their host plants [96,97]; however, the real producers of some known NPs from the plant microbiome or complex assemblages remain a mystery. Fortunately, MSI has been used successfully to reveal the hidden producers of some NPs. In 2010, Taniguchi et al. discovered and characterized a novel depsipeptide, palmyramide A (Figure 3), in the mixture extraction of marine cyanobacterium and a red alga (*Rhodophyta*) [98]. Using MALDI-MSI analysis, it was indicated that the true producer of this new compound was from marine cyanobacterium, *Lyngbya majuscule* [98]. In 2014, Waters et al. proved *Micromonospora* sp. from sponge tissue as the producer of antimalarial natural product, manzamine A (Figure 3), by MALDI-MSI [99]. Another successful case is that the root-associated endophytic microorganism was confirmed to be the hidden producer of maytansine in *Putterlickia* roots using MALDI-MSI [100]. The latest example is the study of cyclic depsipeptide FR900359 on the surface of an *Ardisia crenata* leaf by MALDI-MSI, indicating that the real producer of this bioactive compound may not be from the plant but from the symbiotic bacterium [97].

For most known marine NPs, we rarely know about their real biosynthetic origins. In 2008, Simmons et al. identified the biosynthetic origins of 13-demethylisodysidenin, a previously mysterious natural product, from a thin section of sponge cyanobacterium tissue, using the combined imaging of epifluorescence microscopy and MALDI-MSI, which clearly demonstrated the power of MSI in localizing a specific natural product from complex mixed marine microbial samples [6]. In 2011, during the study of the natural product biosynthesis process in marine cyanobacteria *Lyngbya* strains, Esquenazi et al. offered some new insights into the biosynthetic timing for bromination of Jamaicamide A, with MALDI imaging and stable isotope (^15^N) labeling [101]. In 2012, Xu et al. unraveled the maturation process of an anticancer compound, didemnin B (Figure 3), from the marine α-proteobacteria, *Tistrella mobilis*, by monitoring the time-course production using MALDI-MSI [102]. These examples fully exhibited the considerable capability of MSI in the study of the real biosynthetic origins of marine NPs.

### 4.3. Uncovering the NP-Mediated Microbial Interaction

MSI allows further evaluation of the ecological function of NPs in situ, which is helpful for our understanding of the complicated metabolic interactions between/among different microbes (Table 5 and Table 6). The first impressive application in this aspect is the research of the microbiome on the barnacle surface; Yang et al. identified a peptide from *Promicromonosporaceae* strain SIO-11, with the interesting ecological function of preventing neighboring microorganisms’ migration and a hydroxamate siderophore produced by interspecific competing for iron ions. In this research, after the observation of an obvious microbial interaction on the agar plate, they carefully monitored the time-course MSI data of the microbial single culture and the coculture. The compounds involved were obtained by the purification of the scaled coculture with 600 agar plates. To determine the structure, de novo sequencing was combined with NMR spectroscopic interpretation. This research directly linked the marine microbiome chemotypes to its phenotypes, which provided a paradigm for the investigation of the signal molecules involved in microbial metabolic exchange [103]. In 2012, Andras et al. found that Neurymenolide A, produced by red alga *Phacelocarpus neurymenioides*, could transfer to coral *Porites rus* by physical contact, which led to coral bleaching. In this study, the visualized and quantified result from DESI-MSI indicated the highest concentrations of Neurymenolide A on the blades [104]. In 2013, in their research of sacoglossan–alga association, Davis et al. detected the presence of antifungal compound kahalalide F on a longitudinal section of the sacoglossan *Elysia rufescens* with MALDI-MSI [105]. In 2014, Moree et al. observed *Pseudoalteromonas* sp. OT59, isolated from healthy coral *Leptogorgia alba,* could inhibit marine fungus *P. citrinum*. Then, through monitoring the interaction between the coral and fungus, they discovered the light-dependent antifungal Alteramides (Figure 3) from *Pseudoalteromonas* sp., with the combination of MALDI-MSI and the molecular network [106]. In 2020, Krespach et al. reported that *Streptomyces iranensis* would specifically release algicidal compound Azalomycin F when contacted with *Chlamydomonas reinhardtii,* using MALDI-MSI. With the cooperation of *A. nidulans*, fungal mycelium can protect *Chlamydomonas reinhardtii* from inhabitation by bacteria. This result showed the alga–fungus consortia response to harmful invaders and offered us a better understanding of microbial symbiosis [107]. In 2021, the same research group discovered that when treated with sublethal concentrations of Azalomycin F, *C. reinhardtii* would be induced to form the multicellular structure “gloeocapsoid”, an undescribed self-protective aggregate structure. The formations of algal gloeocapsoids triggered by the other two bacterial marginolactones, Desertomycin A and Monazomycin, were also characterized by MSI and light microscopy [108]. It has been suggested that complex microbiome or microbial interactions could be the real producer of specific NPs [1]; however, the mechanism of microbial response for the inhibition of NPs produced by the competitor is complicated and sophisticated. Tracking the bioactive NPs using MSI in the complicated microbial dynamic metabolic exchanges has gradually confirmed this hypothesis. With MALDI-MSI, scientists have revealed that different microbes can utilize a similar strategy (esterification) to specifically inactivate siderophore Pyochelin from a neighboring competitor for their increasing survival [109,110].

## 5. Current Challenges and Prospects for MSI in Microbial NP Research

From traditional “activity-guided” strategy to combining genomics, bioinformatics and imaging techniques to reveal cryptic and bioactive NPs, a new golden age of microbial NPs is approaching. Benefiting from its splendid qualitative analysis, MSI technology has made great progress in the characterization and identification of NPs from microbes or microbiomes in the past decades. Despite the successful cases, some remaining challenges still need to be surmounted:

(1) With the increasing accessibility of microbial MSI, researchers need to analyze their acquired data carefully, particularly an untargeted approach, peak annotation and deduplication are necessary. Although numerous databases and powerful software are available, careful artificial interpretation of mass spectra is inevitable to avoid missing some cryptic and hidden NPs in microbial metabolic interactions. At present, combined with the molecular network, the localization and identification of known NPs using MSI are achievable. But for unknown NPs, structure elucidation may need high-resolution MS^n^ spectra and 2D-NMR data.
(2) As the most mature and commercial platform, MALDI-MSI has been applied widely in NP research. However due to the background signals from the organic acid matrix, the identification of some small molecules (<500 Da) remains difficult. As a supplementary means, “molecular imprinting techniques” may lose some of the information of the original samples. Thus, it requires the joint efforts of biologists, chemists and materials scientists to explore new matrices with low background interference.
(3) Though sample preparation does not now seem a real challenge in MSI for microbial NP research, it is still the most critical factor, especially for microbes from special habitats such as marine habitats. Due to some unique properties of marine microbes, imaging for those specific samples is still a challenge because of the higher salinity of the media. In order to avoid possible ion formation suppression, it is necessary to optimize the cultural media during sample preparation.
(4) In addition to qualitative analysis, the in situ quantitative imaging of specific metabolites is meaningful. However, only a few attempts have been reported in the field of microbial quantitative MSI [93,141,142]. An uneven microbial sample surface and potential matrix effects, coupled with the different ionization efficiency of the sample morphology, will affect the accuracy and reproducibility of the quantitative MSI results of microbial samples; thus, new breakthrough technologies for exploration are required in this field.

As a promising strategy for microbial MSI, multimodel imaging, which combines microscopy and MSI, has been reported [72]. This technique provides the probe of nano-DESI with a pair of “microscopic eyes” for topography recognition and automated movement. The other case is (MA)LDI-FISH, which combined MALDI-MSI with fluorescence in situ hybridization (FISH) to monitor the antibiotic production from symbiotic bacteria on the cocoons of beewolf wasps [143]. Recently, nontargeted MALDI-LESA MS^2^I has also been applied for confident identification and localization [144]. Ion mobility spectrometry (IMS) has been introduced to couple with MSI [145], which is useful in the identification of isomeric molecules. All these suggest that the combination of multiple techniques and strategies is important for the identification and elucidation of microbial NPs observed in MSI.

In addition to the identification and localization of microbial NPs, we need to broaden our horizon to extremophiles and the complex chemical interactions in microbial communities with MSI. Marine organisms have been proven to be treasure troves for bioactive NPs [146], and their prominent biosynthetic potential has recently been revealed [147]. In recent years, marine microbial NPs keep bringing us surprise [148,149,150]. MSI analysis of some complex marine symbionts, such as sponge microbes, has provided the correlation between the production of NPs and symbiotic microbes [151]. However, the lower sample accessibility is one of the most crucial challenges in marine NP study, especially for deep-sea microbes. Therefore, label-free, high-resolution, in situ and untargeted MSI technology seems irreplaceable in the study of samples’ NPs from extreme environments. Since many fields have benefited from MSI, we wish that in the next few years it can become a routine weapon for marine microbial NP exploration, to extend the understanding of the molecular basis of interactions between different marine organisms and the real chemical ecological functions of NPs in marine ecosystems.

## Figures and Tables

**Table 1:** Ionization techniques used in microbial MSI.

Ion Source Working Condition	Ionization Source	Limit of Spatial Resolution	Advantage	Major Limit
Vacuum ionization	SIMS	35 nm [23]	Superior spatial resolution	Mostly fragmentation ions
	MALDI	5 μm [7]	Broad coverage of molecule species	The choice of matrix should be considered
	SALDI	10 μm [7]	Matrix-free	Sample transfer is inevitable
Ambient ionization	DESI	10 μm [24,25]	Simple sample preparation	Complex instrumental parameters optimization
	LAESI	100 μm [26]	Can be used for fresh samples	Lower spatial resolution

Note: SIMS: secondary ion mass spectrometry; MALDI: matrix-assisted laser desorption/ionization; SALDI: surface-assisted laser desorption/ionization; DESI: desorption electrospray ionization; LAESI: laser ablation electrospray ionization.

**Table 2:** Technical advances in sample preparation of microbial MALDI-MSI.

Time	Introduced Technique	Advantage	Reference
2012	A common protocol for agar-based microbial MALDI-MSI	The first protocol for agar-based microbial MALDI-MSI	[61]
2013	A method for visible three-dimensional (3D) models of a microbial colony	Captures the depth profile of metabolite distribution beyond 2D-MALDI-MSI	[63]
2014	A method with solid MALDI matrix deposition on microbial agar culture	Enhanced signals of some fungal metabolites	[64]
2015	A method for spraying matrix solution programmatically on dried agar-based samples	Forms a homogeneous, evenly closed matrix layer	[65]
2016	A robotic matrix sprayer with a heated capillary	Higher sensitivity and lateral resolution for the analyte and suitable for different kinds of matrix	[66]
2016	A one-step matrix spraying method with optimized homemade equipment	Simplified sample preparation and high-resolved images	[67]
2019	A membrane-based culturing workflow	Offers a safe and flat microbial sample surface	[68]
2022	A method using 2,5-dihydroxybenzoic acid (DHB) as “glue” to adhere the microbial culture agar plate to the MALDI target	Prevents the sample flaking from the target under vacuum and also provides a larger area for MALDI-MSI analysis	[35]

Note: MALDI: matrix-assisted laser desorption/ionization; MSI: mass spectrometry imaging.

**Table 3:** Technical advances in sample preparation of microbial DESI-MSI.

Time	Introduced Technique	Advantage	Reference
2010	A thin film imprinting technique with mixed cellulose ester filter membranes	Uses a complementary surface to make an imprint of the bacterial culture from solid agar for imaging	[57]
2012	Nano-DESI-MSI technology	Direct chemical monitoring of living microbial colonies grown on a Petri dish	[70]
2014	A “cardboard insert” method	An effective method for fungal culture imaging in situ with a hard and flat surface	[71]
2015	A protocol on microbial agar culture for direct DESI-MSI	Offers rapid sample preparation with a dehydrated and hard surface for imaging	[69]
2017	A constant-distance nano-DESI-MSI imaging model	An ideal method for imaging microbial samples with complex topography	[72]
2019	A microbial sample reparation method with microporous membrane scaffolds (MMS)	An effective method for imaging and evaluating microbial interspecies interactions	[73]

Note: DESI: desorption electrospray ionization; MSI: mass spectrometry imaging.

**Table 5:** MSI application in the research of microbial binary interaction.

Species	Ionization Method	Compound	Research Purpose	Reference
*Bacillus subtilis* vs. *Streptomyces coelicolor*	MALDI	Surfactin, plipastatin, Prodiginines, CDA, SapB	Characterization of bacterial metabolic exchange	[111]
*Bacillus subtilis* vs. *Streptomyces coelicolor*	DESI	Actinorhodin, surfactin, plipastatin	Bacterial interaction	[57]
*Bacillus subtilis*	MALDI	Sporulation-delaying protein (SDP), sporulation killing factor (SKF)	Intraspecies interaction	[112]
*Streptomyces roseosporus* vs. *Streptomyces epidermidis*	MALDI	Arylomycins	Discovery of bioactive NPs	[74]
*Bacillus subtilis* vs. *Staphylococcus aureus*	MALDI	Surfactin, plipastatin, PSMλ, PSMα3	Microbial competition	[113]
*Streptomyces* sp. Mg1 vs. *Bacillus subtilis*	MALDI	Polyglutamate, surfactin, plipastatin, chalcomycin A	Microbial competition	[114]
*Streptomyces* sp. Mg1 vs. *Bacillus subtilis*	MALDI	Surfactin, hydrolyzed surfactin	Interspecies competition	[115]
*Streptomyces coelicolor* vs. other five species actinomycetes	MALDI and nano-DESI	Acyl-desferrioxamine siderophores	Interspecies interaction	[116]
Endophytic bacteria of *Cannabis sativa L* vs. *Chromobacterium Violaceum*	MALDI	N-acylated L-homoserine lactones (AHLs)	Quorum sensing	[117]
*Pseudomonas aeruginosa* vs. *Staphylococcus aureus*	MALDI	Pyocyanin, rhamnolipid, 4-hydroxy-2-alkylquinoline (HAQ) derivatives	Metabolic profile and interspecies interaction	[118]
*Paraconiothyrium variabile* vs. *Bacillus subtilis*	MALDI	Surfactin	Investigation of antagonism	[119]
*Paenibacillus dendritiformis* vs. *Bacillus subtilis* NCIB3610	MALDI	Surfactin and its degradations	Specific interaction of attractant	[120]
*Myxococcus xanthus* DK1622 vs. *Escherichia coli*	DESI	Myxovirescin A, DKxanthene-560	Investigation of predation process	[73]
*Janthinobacterium agaricidamnosum* vs. *Agaricus bisporus*	MALDI	Jagaricin A	Investigation of the virulence factors of soft rot bacteria	[121]
*Pseudomonas aeruginosa* vs. *Aspergillus fumigatus*	MALDI	Fungal siderophores, phenazine metabolites	Interkingdom metabolic transformation	[122]
*Paenibacillus polymyxa* (Pp56) vs. *Fusarium oxysporum*	MALDI	Fusaricidins A, B, C, lipopeptides	Searching for microbial biocontrol agents	[123]
*Fusarium solani* vs. *Achromobacter xylosoxidans*	MALDI	Hexacyclopeptides	Discovering metabolites produced by endophytes	[124]
*Ralstonia solanacearum* vs. *Aspergillus flavus*	MALDI	Ralsolamycin	Interkingdom interaction	[125]
*Burkholderia seminalis* vs. *Moniliophthora perniciosa, Phytophthora capsisi, Phytophthora palmivora* and *Phytophthora citrophtora*	DESI	Phospholipids, rhamnolipid	Searching for cacao pathogens’ biocontrol agents	[126]
*Streptomyces* sp. (CB0028) vs. *Escovopsis* sp. (CBAcro424)	MALDI	Siderophores	Searching for natural products in microbial interaction	[127]
*Ralstonia solanacearum* vs*. Fusarium fujikuroi* and *Botrytis cinerea*	MALDI	Ralsolamycin, bikaverin	Small molecular induced microbial interaction	[128]
*Burkholderia cenocepacia* 869T2 vs. *Phellinus noxius*	MALDI	Pyochelin and its esterification product	Dynamic changes of metabolites in microbial interactions	[109]
*Staphylococcus aureus* vs. *Pseudomonas aeruginosa*	MALDI	Pyochelin, pyochelin methylester	Bacterial competition in vivo	[110]
*Trichoderma harzianum* vs. *Moniliophthora roreri*	DESI	T39 butenolide, harzianolide, sorbicillinol	Investigation of antagonistic interaction of fungi	[129]
*Phellinus noxius* vs. *Aspergillus* sp. 3Y and 3G	SALDI	Sterigmatocystin, fellutamides	Developing microbial MSI on nanostructured silicon	[54]
*Penicillium polonicum* ACCC31573 vs. *Fusarium oxysporum f.* sp*. lycopersici* 4287	MALDI	Fructigenine A and B	Screening for antifungal antibiotics	[130]
*Penicillium digitatum* vs. *Penicillium citrinum*	DESI	Tryptoquialanines, 15-dimethyl-2-epi-fumiquinazoline A, deoxytryptoquialanone, citrinadin A, deoxycitrinadin A, chrysogenamide A, tetrapeptide	Screening for new antifungal compounds	[131]
*Purpureocillium lilacinum* vs. *Botrytis cinerea*	MALDI	Leucinostatin Z	Searching for new natural products	[132]

Note: DESI: desorption electrospray ionization; MALDI: matrix-assisted laser desorption/ionization; SALDI: surface-assisted laser desorption/ionization.

**Table 6:** MSI application in the research of complex microbial communities or symbionts.

Imaging Sample	Ionization Method	Compound	Research Purpose	Reference
Beewolf	MALDI	Streptochlorin and eight piericidin derivatives	Beewolf–Streptomyces symbiosis	[133]
Leaf-cutting ant	MALDI	Valinomycin	Ecological role of microorganisms associated with leaf-cutting ants	[134]
Ant-derived Streptomyces spp. and Escovopsis spp.	MALDI	Actinomycins D, actinomycins X2, actinomycins X0β, elaiophylin, efomycin A, efomycin G, shearinines D, F and J	Chemistry-based microbial interactions in ant microbe symbiosis	[135]
Pseudonocardia on the surface of ants’ propleural plates	MALDI	Ergothioneine	Visualization of bacterial-derived compounds on the ant exoskeleton	[136]
Frog skin associated bacteria Pseudomonas cichorii	MALDI	Viscosin-like lipopeptides	Searching for antifungal compounds	[137]
Plant roots colonized by Bacillus amyloliquefaciens S499	MALDI	Surfactins, iturins, fengycins	Investigation of plant–microbe interactions at the molecular level	[138]
Putterlickia verrucosa root	MALDI	Maytansine	Searching for the real producer of maytansine	[100]
Lichen	MALDI	Pyridone alkaloid, asperphenamate, alantolactone, mannitol, polysaccharide-containing mannitol, pheophorbide A, pheophytin A	Show the chemical diversity and distribution of the microbial community of a Peltigera lichen	[139]
Arabidopsis leaf-derived bacteria	MALDI	Streptocidin A and D, phosphobrevin, marthiapeptide A, macrobrevin	Antibiotic production potential of the *Arabidopsis* leaf microbiome	[77]
Root nodule	MALDI	Britacidins, tyrocidines	The research of specific metabolites from Brevibacillus brevis	[140]

Note: MALDI: matrix-assisted laser desorption/ionization.

## Data Availability

No new data were created or analyzed in this study. Data sharing is not applicable to this article.

## Abbreviations

AP	Atmospheric pressure
DESI	Desorption electrospray ionization
ESI	Electrospray ionization
FT-ICR	Fourier transform ion cyclotron resonance
HR	High resolution
LAESI	Laser ablation electrospray ionization
MALDI	Matrix-assisted laser desorption/ionization
MSI	Mass spectrometry imaging (also known as IMS in other papers)
NMR	Nuclear magnetic resonance
NPs	Natural products
Q	Quadrupole
SALDI	Surface-assisted laser desorption/ionization
SIMS	Secondary ion mass spectrometry
TOF	Time-of-flight
